# The 5, 10 methylenetetrahydrofolate reductase C677T mutation and risk of fetal loss: a case series and review of the literature

**DOI:** 10.1186/1477-9560-5-17

**Published:** 2007-10-17

**Authors:** Ivy Altomare, Alan Adler, Louis M Aledort

**Affiliations:** 1 Mount Sinai Medical Center, 1 Gustave L. Levy Pl., New York, USA

## Abstract

**Background:**

The true relationship between methylenetetrahydrofolate reductase C677T homozygosity and risk of recurrent spontaneous abortion is unknown, and it is unclear if women with these mutations should be anticoagulated during pregnancy.

**Objectives:**

We report a series of 8 patients with this issue and review the current literature.

**Methods:**

8 patients (3 of whom were actively pregnant) were referred with histories of spontaneous fetal loss; hypercoaguability work-ups revealed each were homozygous for the MTHFR C677T mutation without other thrombophilias.

**Results:**

In the 3 women who have conceived, treatment with LMW heparin during pregnancy led to two full-term births and one additional pregnancy without complication. For the 5 who have not, we recommended treatment with LMW heparin upon conception.

**Conclusion:**

We provide evidence to support the relationship between MTHFR C677T mutations and recurrent fetal loss, and to suggest that anticoagulation of these patients during pregnancy can lead to a successful pregnancy outcome.

## Background

There is growing evidence that thrombophilia defects can impact pregnancy outcomes. It is already well-known that the acquired thrombophilia of the antiphospholipid syndrome, seen with lupus anticoagulant and/or anticardiolipin antibody seropositivity, is strongly associated with recurrent fetal loss[1]. In the literature, various hereditary thrombophilias such as activated protein C resistance, the factor V leiden mutation (both homozygosity and heterozygosity), the prothrombin G20210A mutation, protein S deficiency, the methylenetetrahydrofolate reductase (MTHFR) C677T mutation, hyperhomocysteinemia, or combinations of the above disorders have also been linked to pregnancy loss at varying stages of gestation [2,3]. Yet because many of the published case series and meta-analyses describe conflicting results, the relationship between a MTHFR mutation and recurrent miscarriages remains confusing. We present eight cases from our institution that may well represent the impact of homozygosity of the MTHFR C677T mutation on early pregnancy loss.

## Case series

Eight women presented within a span of three months to our institution with histories of one or more miscarriages after the 10^th ^week of gestation. These women were referred to our program after their obstetricians had excluded common causes of fetal loss. None of these women had TORCH infections or gestational diabetes, and karyotype analyses on the products of conception (when possible) were normal. We were asked, in each case, to evaluate for thrombophilia as a cause of recurrent miscarriage and to determine if intervention (anticoagulation) would help to prevent future miscarriages. Each of these women denied a personal or family history of thrombosis.

For each patient, blood tests for both genetic and acquired causes of thrombophilia were performed (see Table 1). Pregnancy loss after the 10^th ^week of gestation has been more closely associated with thrombophilia, as opposed to earlier losses in weeks 3 to 9[4]. The mean age of these women was 36 years, and the number of previous miscarriages ranged from 1 to 6. The clinical features of each case and the results of the thrombophilia work-ups are reported in Table 2. Each patient was found to be homozygous for the MTHFR C677T mutation. One patient was also heterozygous for the MTHFR A1298C mutation, three were heterozygous for the PAI-1 gene polymorphism 4G/5G, and one was homozygous for the PAI-1 gene polymorphism 4G/4G. Not surprisingly, homocysteine levels were in normal range for each patient, as they were each taking prenatal vitamins.

**Table 1:** Hypercoaguability Profile

Fibrinogen
Factor VIII
Factor IX
Factor XI
Anti-Phosphatidyl Serine IgG
β2 Glycoprotein
Anti-Cardiolipin IgM and IgG
Lupus anticoagulant
Plasminogen
Euglobulin clot lysis
Homocysteine
Factor V Leiden
Prothrombin G20210A allele
Protein C
Protein S
Antithrombin III
5,10 MTHFR gene
PAI-1 polymorphism gene
Activated Protein C resistance

**Table 2:** Patient Data

Patient	Age	Miscarriages	Normal births	Defect	Outcome on LMW heparin
1	27	2 in first trimester 1 in second trimester	1	MTHFR C677T homozygous A1298C heterozygous	N/A
2	41	2 in first trimester	0	MTHFR C677T homozygous	N/A
3	39	2 in first trimester	1	MTHFR C677T homozygous PAI-1 heterozygous 4G/5G	Pregnancy with successful delivery
4	44	2 in first trimester	0	MTHFR C677T homozygous	Pregnancy with successful delivery
5	27	1 in first trimester	3	MTHFR C677T homozygous PAI-1 heterozygous 4G/5G	N/A
6	34	6 in first trimester	3	MTHFR C677T homozygous PAI-1 heterozygous 4G/5G	Pregnant, in third trimester
7	32	2 in first trimester	2	MTHFR C677T homozygous	N/A
8	40	3 in first trimester	0	MTHFR C677T homozygous PAI-1 homozygous 4G/4G	N/A

We reviewed the literature in order to find evidence to support or refute the association between MTHFR mutations and adverse pregnancy outcomes, and to guide us in managing these patients. Patients 1, 2, 3, 7 and 8 were seen hoping to become pregnant again, while patients 4, 5 and 6 were already pregnant and not as yet anticoagulated.

## Results

We recommended treatment doses of low-molecular weight (LMW) heparin as a therapeutic intervention in hopes of achieving a full-term pregnancy and reducing the risk of recurrent abortion for future pregnancies. Patients 4 and 6 who were pregnant received LMW heparin immediately. Unfortunately, patient 5 miscarried prior to starting LMW heparin, but was able to conceive again and received LMW heparin shortly after this next conception. We recommended LMW heparin upon conception for patients 1, 2, 3, 7 and 8 who were not yet pregnant. As seen in Table 2, two women had successful full-term deliveries on LMW heparin, while a third woman is currently pregnant (in her third trimester) and has been taking LMW heparin since conception. Five patients are trying to conceive.

## Discussion

Methyltetrahydrofolate reductase is an enzyme which catalyzes the reduction of 5, 10-methyltetrahydrofolate and allows the re-methylation of homocysteine to methionine [5]. A variant form of 5-MTHFR, termed "thermolabile" because of poor heat stability *in vitro*, was first described in 1988, and was found to be associated with the development of coronary artery disease. Homozygosity for the gene encoding this thermolabile mutant provides only 50% of normal MTHFR enzyme activity, and can lead to hyperhomocysteinemia [6]. Over the past two decades, multiple reports have cited the MTHFR mutation as a risk factor for both thrombosis and pregnancy loss, while others have refuted the association. Exactly how MTHFR mutations can cause pregnancy complications is unknown; a logical hypothesis is that endothelial damage from hyperhomocysteinemia leads to venous thromboemboli and placental insufficiency. In a retrospective study of 24 Indian women with recurrent pregnancy loss, the highest values of homocysteine were found in those who carried the MTHFR gene[7]. However, other reports refute this hypothesis. Another study compared homocysteine levels in 57 pregnant women with and without the MTHFR C677T mutation and found that homocysteine concentrations were not different by MTHFR genotype[8], suggesting that fetal loss associated with the MTHFR mutation can be seen in the absence of high homocysteine levels. Indeed, this is what we saw in our four patients, who are homozygous for the C677T mutation and have had several miscarriages, yet their homocysteine levels were always normal. Therefore, a different mechanism for fetal loss, less dependant on hyperhomocysteinemia, may exist.

Interestingly, patient 1 described an episode of transient spontaneous bilateral hearing loss seven years prior. Transient hearing loss can be caused by vascular insufficiency and is associated with MTHFR mutations [9]. Of note, patient #2 is additionally heterozygous for another MTHFR mutation, A1298C, the second most common mutant polymorphism of the MTHFR mutant gene. It is estimated that 15–20% of the general population are heterozygous for one of the two variants, with prevalence even higher in certain ethnicities[10]. However, the A1298C polymorphism has never been reported as a cause of recurrent fetal loss, and has no role in causing thrombophilia [11].

MTHFR mutations have been linked to various adverse pregnancy outcomes: specifically, early fetal loss (most commonly defined as spontaneous abortion in the first or second trimester), late fetal loss (death in the third trimester), preecclampsia, intrauterine growth retardation, placental abruption and neural tube defects. We found it difficult to find clear evidence for or against the association between MTHFR mutations and the pregnancy outcomes listed above. Much of the data supporting a causative association is in the form of isolated case reports and small case series, which have been excluded from the larger meta-analyses discussed below[2,12,13]. Also, the definition of recurrent fetal loss varies widely among studies with respect to number of previous losses (1 to 3), the timing of losses (consecutive vs. non), and the age of gestational loss (less than 12 weeks through less than 24 weeks), making it difficult to interpret the data and compare the studies. There are a number of retrospective and case-control studies demonstrating a significantly higher frequency of MTHFR mutations in patients with recurrent fetal loss than in the control groups [14-16]. An often-cited study supporting the role of MTHFR mutations in recurrent unexplained abortions found a 2–3 fold increased risk of early fetal loss among 185 Caucasian females homozygous for the C677T mutation versus 113 normal controls[17]. These studies, combined with numerous case reports, strongly suggest a significant association between recurrent miscarriage and the mutant MTHFR allele.

Recent meta-analyses have pooled several of these and other studies, and concluded that in most cases the relationship between MTHFR mutations and adverse pregnancy outcomes is not truly significant. One meta-analysis of ten studies specifically addressing MTHFR mutations and hyperhomocysteinemia found an odds ratio of 1.4 favoring higher risk of recurrent pregnancy loss, but the confidence interval was 1.0–2.0[12]. A second meta-analysis pooled eight studies of recurrent and six of non-recurrent fetal loss, and found no association between homozygous MTHFR mutations and either clinical outcome [13]. And finally, the third and largest meta-analysis of thrombophilia in pregnancy found no association between MTHFR mutant homozygosity and venous thromboembolism or recurrent first trimester loss. There was a trend toward higher risk in the pooled analyses for non-recurrent early loss and late loss, but both confidence intervals crossed the one. This meta-analysis did find that patients with MTHFR mutant homozygosity are at increased risk of pre-ecclampsia, with a odds ratio of 1.37 reaching statistical significance [2].

Given the above data, we as clinicians are left with a dilemma of whether or not to recommend anticoagulation for patients such as these. Again, we feel that definitive evidence for or against the association of MTHFR mutations and adverse pregnancy outcomes is lacking. Admittedly, there are limitations of meta-analyses as well as the smaller positive studies. Many published case reports describe successful pregnancy outcomes with the prophylactic use of anticoagulation (most commonly low-molecular weight heparin) in women with MTHFR mutations and multiple recurrent miscarriages. However, no randomized studies exist to address the use of anticoagulation in pregnant women with MTHFR mutations. We found one study in which 351 women with recurrent miscarriages (12% with the MTHFR mutation) were treated with prophylactic asprin + unfractionated or LMW heparin to term; the success rate of normal term delivery in this study was 94% [18].

Treatment of 3 patients with LMW heparin during pregnancy led to successful deliveries in two women, while another woman is pregnant in her third trimester with no complications thus far and is scheduled for induction. We plan to treat patients 1, 2, 5, 7 and 8 with LMW heparin once they become pregnant. An eighth patient with no history of fetal loss presented in consultation after a hypercoaguable work-up was performed prior to the planned use of hormonal agents to treat infertility (not detailed). She too is homozygous for the MTHFR mutation, in addition to being a carrier of the PAI-1 polymorphism. We recommended anticoagulation for this patient as well.

## Conclusion

Although our report is also a case series, we feel it adds to the literature that the relationship between MTHFR C677T homozygosity and spontaneous abortions may be real, and we are encouraged that treatment with LMW heparin appears to have led to two successful deliveries and one uncomplicated near-complete pregnancy thus far. Only this kind of surveillance and reporting will establish whether this relationship will hold up over time.

## Competing interests

The author(s) declare that they have no competing interests.
